# Machine learning-driven prediction of cycloplegic refractive error in Chinese children

**DOI:** 10.3389/fcell.2025.1608494

**Published:** 2025-05-22

**Authors:** Bichi Chen, Li Tian, Fuyue Tian, Qiaochu Yang, Ying Ruan, Ying Li, Min Cao, Chuanyan Wu, Maoyuan Yang, Suzhong Xu, Ruzhi Deng

**Affiliations:** ^1^ Vision X Medical Technology Co., Ltd., Shanghai, China; ^2^ National Clinical Research Center for Ocular Diseases, Eye Hospital, Wenzhou Medical University, Wenzhou, China; ^3^ Shanghai E-Vision Eye Clinic, Shanghai, China

**Keywords:** refractive error, prediction, machine learning, cycloplegic refraction, myopia

## Abstract

**Objective:**

To develop and validate machine learning (ML) models for predicting cycloplegic spherical equivalent refraction (SER) using non-cycloplegic parameters, addressing challenges in pediatric ophthalmic assessments.

**Methods:**

A prospective cohort of 2,274 Chinese children (4,548 eyes) aged 3∼16 years was stratified into development (*n* = 1819) and validation (*n* = 455) datasets. Six ML models (linear regression, random forest, extreme gradient boosting, multilayer perceptron, support vector machine, and light gradient boosting machine) were trained on demographics, non-cycloplegic refractive error, and ocular biometrics. Model performance was evaluated using *R* ^*2*^, mean error (ME), mean absolute error (MAE), and clinical accuracy (proportions within ±0.50 D/±1.00 D).

**Results:**

In the validation dataset, ML models predicted cycloplegic SER with high *R* ^*2*^ (0.920∼0.934), low ME (−0.004∼0.015 D) and MAE (0.385∼0.413 D). The multilayer perceptron model achieved the highest accuracy (*R* ^*2*^ = 0.934, MAE = 0.385 D), with 73.08% and 94.29% of predictions within ±0.50 D and ±1.00 D, respectively. Performance was optimal in children aged 7∼10 years (77.17∼79.70% within ±0.50 D) and those with low myopia (−3.00 to −0.50 D; 83.09∼83.56% within ±0.50 D). Non-cycloplegic measurements systematically overestimated myopia (mean difference: −0.39 ± 0.71 D, *P* < 0.001), particularly in younger children and hyperopic eyes.

**Conclusion:**

ML models provide accurate estimates of cycloplegic SER using non-cycloplegic parameters, offering a practical alternative for pediatric refractive assessments when cycloplegia is infeasible.

## 1 Introduction

Accurate refractive error assessment in pediatric populations necessitates cycloplegic refraction to eliminate accommodation-induced measurement errors, establishing it as the clinical gold standard for both diagnosis and epidemiological studies (Morgan et al., 2015; Doherty et al., 2019; Flitcroft et al., 2019). However, application of cycloplegia faces practical challenges in large-scale pediatric research, including time constraints, resource limitations, and potential contraindications, often precluding its routine use in population-based screenings. To address the conflict between the necessity of cycloplegic refractive examination and the difficulty of performing cycloplegia in practice, emerging studies have explored statistical models to predict cycloplegic refractive error using non-cycloplegic parameters. These models exhibit inconsistent performance (*R* ^*2*^ ranging from 0.26 to 0.935) (Ip et al., 2007; Kimura et al., 2007; He et al., 2015; Foo et al., 2016; Sankaridurg et al., 2017; Magome et al., 2021; Wang et al., 2022; Du et al., 2023; He et al., 2023; Ying et al., 2024), likely attributable to methodological heterogeneity, variable predictor selection, and population-specific biases.

Machine learning (ML), a branch of artificial intelligence, offers unparalleled capabilities in identifying complex patterns within high-dimensional datasets. Unlike conventional regression models constrained by linear assumptions, advanced ML algorithms (e.g., random forest [RF], support vector machine [SVM] and gradient boosting models) are capable of modeling intricate and non-linear relationships between predictors and outcomes, and have demonstrated great potential for application in ophthalmology and vision research (Wu et al., 2021; Cao et al., 2022; Wu et al., 2022; Chandra and Ying, 2023; 2024). Recent advancements highlight ML’s ability to predict key refractive parameters such as spherical equivalent refraction (SER) and axial length (AL) in pediatric cohorts. For instance, Zhu et al. (2023) demonstrated that ML models could predict SER and AL changes in children with high accuracy (*R* ^*2*^ up to 0.90), leveraging longitudinal data to inform myopia progression. These studies collectively emphasize ML’s capacity to address both diagnostic and prognostic challenges in pediatric ophthalmology, particularly when traditional methods are constrained.

This study aimed to develop and validate ML models for predicting cycloplegic refractive error in Chinese children aged 3–16 years old using readily obtainable non-cycloplegic parameters, encompassing demographics, non-cycloplegic refractive error, and ocular biometric measurements. By leveraging the predictive power of ML, this work seeks to provide a clinically practical solution for determining true refractive status in pediatric populations where cycloplegic examinations are impractical.

## 2 Materials and methods

### 2.1 Participants

A prospective cohort of 2,274 children (4,548 eyes) aged 3–16 years was recruited from the Eye Hospital of Wenzhou Medical University between October 2021 and March 2024. Inclusion criteria included: (1) parental consent for cycloplegic procedures; (2) availability of pre-/post-cycloplegic clinical data. Exclusion criteria comprised ocular organic diseases, strabismus, amblyopia, prior ocular surgery, or hypersensitivity to 1% cyclopentolate hydrochloride. Participants were stratified by age and refractive status and randomly allocated into development (80%, *n* = 1819 children) and validation (20%, *n* = 455 children) datasets to ensure proportional representation.

The study protocol was approved by the Institutional Ethics Committee of the Eye Hospital of Wenzhou Medical University (approval no. 2021-233-K-203-03) and adhered to the tenets of the Declaration of Helsinki. Written informed consent was obtained from all participants’ parents or guardians.

### 2.2 Data collection

Demographic information (age, gender, and height) and ocular parameters were collected under non-cycloplegic conditions. Comprehensive ophthalmic examinations included: distance visual acuity test using retro-illuminated logMAR charts with tumbling-E optotypes, slit-lamp examination of the anterior segment, fundus examination with ophthalmoscopy, intraocular pressure measurement and the assessment of ocular biometrics under non-cycloplegic conditions (e.g., AL, corneal curvature [CR], central corneal thickness [CCT], aqueous depth [AD], lens thickness [LT], and corneal astigmatism) using the AB-800 optical biometer (Hangzhou Weixiao Medical Technology Co., Ltd., Hangzhou, China). AL measurements were retained if intra-test variability was ≤0.02 mm.

Cycloplegia was induced by administering two drops of 1% cyclopentolate (Alcon, Fortworth, TX, America) at 5-min intervals. Autorefraction (KR 800, Topcon, Tokyo, Japan) was performed pre-cycloplegia and 30 min after application of cyclopentolate (following confirmation of adequate pupillary dilation [>6 mm] and loss of light reflex). For both conditions, three consecutive autorefraction measurements were averaged, with repeat assessments if any spherical or cylindrical component differed by > 0.50 D between readings. All data underwent dual-entry verification (EpiData 3.1, Odense, Denmark) with source document reconciliation for discrepancies.

### 2.3 Machine learning models

The cycloplegic SER was calculated as sphere +0.5*cylinder. Six ML models were evaluated for cycloplegic SER prediction: (1) linear regression: identifies linear relationships through coefficient optimization, offering interpretability but constrained to additive patterns (Ludwig Fahrmeir and Lang, 2009). (2) RF: combines predictions from an ensemble of decision trees to mitigate overfitting (Breiman, 2001). (3) extreme gradient boosting (XGBoost): utilizes gradient-boosted trees and trained additively to enhance predictive accuracy (Chen and Guestrin, 2016). (4) multilayer perceptron (MLP): a feedforward neural network employing weighted linear summations and nonlinear activation functions for hierarchical feature transformation (Abu-Mostafa and Hsuan-Tien Lin, 2012). The MLP architecture comprised three fully connected layers: an input layer (11 neurons), a hidden layer (32 neurons), and an output layer (1 neuron). (5) SVM: predicts a continuous outcome with high generalization ability by transforming data to high-dimensional spaces (Cortes and Vapnik, 1995). (6) light gradient boosting machine (LightGBM): accelerates gradient boosting via histogram-based tree construction and leaf-wise growth, prioritizing efficiency for large datasets (Ke et al., 2017).

The predictors for the ML models were: demographics (age, gender, height), non-cycloplegic SER, biometric parameters (AL, CR, AL/CR ratio, CCT, AD, LT, and corneal astigmatic value). Hyperparameters were tuned for each of the models through 5-fold cross-validation on the development dataset to reduce overfitting risks and optimize *R* ^*2*^ (the coefficient of determination). Final models were trained on the entire development dataset using optimized hyperparameters. The independent validation dataset was strictly reserved for final performance evaluation, thereby ensuring an unbiased assessment of the models’ generalizability.

### 2.4 Statistical analysis

Myopia was defined as cycloplegic SER -0.50 D or worse. Emmetropia was defined between −0.50 D and +0.50 D and hyperopia was defined as greater than +0.50 D. Continuous variables were expressed as mean ± standard deviation (SD). Paired *t*-test was performed to compare the differences between non-cycloplegic and cycloplegic SER across the entire cohort and in subgroups based on age, refractive error, axial length. Model performance was evaluated using *R* ^*2*^, correlation coefficient *r*, mean error (ME), and mean absolute error (MAE) of differences between predicted and measured cycloplegic SER (calculated as predicted–measured), as well as the clinical accuracy proportions (predictions within ±0.50 D/±1.00 D). The predicted and measured cycloplegic SER were also statistically compared using paired *t*-test. For the best-performing model (selected based on the performance in the validation dataset), subgroup analyses were conducted by age and refractive error magnitude.

ML models implementation utilized Python 3.8 (scikit-learn version 1.3.0), while the statistical analysis was performed with IBM SPSS Statistics software version 27 (IBM, Armonk, NY). A *P* value of <0.05 was considered statistically significant.

## 3 Results

### 3.1 Participant characteristics

Data from 2,274 children (4,548 eyes) were included in the analysis, with a mean age of 7.58 ± 1.94 years (range: 3∼16 years). Females accounted for 50.35% of the study population. Participants were stratified into development (*n* = 1819 children, 3,638 eyes; mean age: 7.58 ± 1.95 years) and validation (*n* = 455 children, 910 eyes; mean age: 7.55 ± 1.92 years) datasets. The two datasets exhibited comparable distributions in age, gender, height, and ocular biometric parameters (all *P* values > 0.05). Demographic and ocular characteristics of participants in the development and validation datasets were summarized in Table 1.

**TABLE 1:** Characteristics of participants in the development and validation datasets.

Characteristics	Development dataset (n = 1819)	Validation dataset (n = 455)	All (n = 2,274)
Age (y), n (%)			
3	24 (1.32)	7 (1.54)	31 (1.36)
4	122 (6.71)	30 (6.59)	152 (6.68)
5	200 (11.00)	50 (10.99)	250 (10.99)
6	348 (19.13)	87 (19.12)	435 (19.13)
7	450 (24.74)	113 (24.84)	563 (24.76)
8	313 (17.21)	78 (17.14)	391 (17.19)
9	159 (8.74)	39 (8.57)	198 (8.71)
10	95 (5.22)	24 (5.27)	119 (5.23)
11	58 (3.19)	14 (3.08)	72 (3.17)
≥12	50 (2.75)	13 (2.86)	63 (2.77)
Mean ± SD	7.58 ± 1.95	7.55 ± 1.92	7.58 ± 1.94
Male: Female (%)	50.25:49.75	47.25:52.75	49.65:50.35
Cycloplegic SER (D), n (%)			
≤−6.0	36 (0.99)	8 (0.88)	44 (0.97)
>−6.0, ≤−3.0	176 (4.84)	46 (5.05)	222 (4.88)
>−3.0, ≤−0.5	1369 (37.63)	343 (37.69)	1712 (37.64)
>−0.5, ≤+0.5	693 (19.05)	169 (18.57)	862 (18.95)
>+0.5, ≤+2.0	956 (26.28)	245 (26.92)	1201 (26.41)
>+2.0	408 (11.21)	99 (10.88)	507 (11.15)
Mean ± SD	−0.04 ± 2.12	−0.05 ± 2.07	−0.04 ± 2.11
Non-cycloplegic SER (D), n (%)			
≤−6.0	40 (1.10)	7 (0.77)	47 (1.03)
>−6.0, ≤−3.0	182 (5.00)	56 (6.15)	238 (5.23)
>−3.0, ≤−0.5	1563 (42.96)	376 (41.32)	1939 (42.63)
>−0.5, ≤+0.5	1149 (31.58)	301 (33.08)	1450 (31.88)
>+0.5, ≤+2.0	489 (13.44)	114 (12.53)	603 (13.26)
>+2.0	215 (5.91)	56 (6.15)	271 (5.96)
Mean ± SD	−0.43 ± 1.86	−0.43 ± 1.84	−0.43 ± 1.85
Height (cm)	126.46 ± 12.68	126.50 ± 13.10	126.47 ± 12.76
Axial length (mm)	23.24 ± 1.13	23.23 ± 1.09	23.23 ± 1.12
Corneal curvature radius (mm)	7.79 ± 0.27	7.79 ± 0.29	7.79 ± 0.27
Corneal astigmatism (D)	−1.38 ± 0.78	−1.40 ± 0.76	−1.39 ± 0.77
Axial length/corneal curvature radius ratio	2.98 ± 0.14	2.98 ± 0.13	2.98 ± 0.14
Central corneal thickness (μm)	537.63 ± 32.23	537.04 ± 32.98	537.51 ± 32.38
Aqueous depth (mm)	2.97 ± 0.30	2.96 ± 0.31	2.97 ± 0.30
Lens thickness (mm)	3.62 ± 0.26	3.63 ± 0.26	3.62 ± 0.26

### 3.2 Mean difference between non-cycloplegic and cycloplegic SER

**FIGURE 1:**
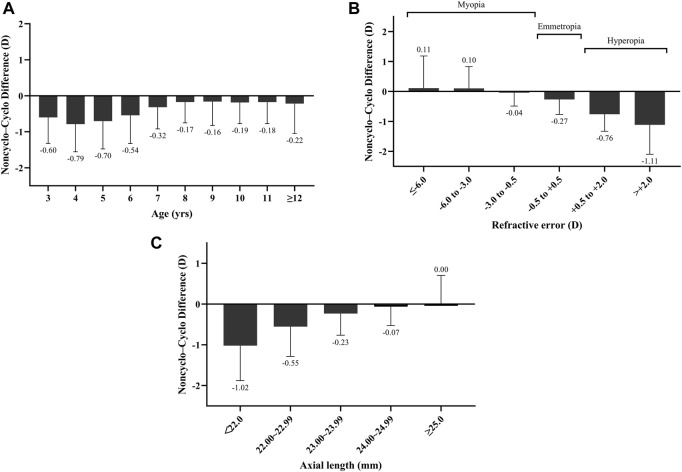
Differences between non-cycloplegic versus cycloplegic refraction based on age **(A)**, refractive error **(B)**, axial length **(C)**.

Overall, there was a mean paired difference of −0.39 ± 0.71 D (95% confidence intervals: −0.41 to −0.37D, *P* < 0.001) between non-cycloplegic and cycloplegic SER, with the non-cycloplegic SER resulting in a more myopic (or less hyperopic) refractive error. Figure 1 illustrated the mean paired differences in non-cycloplegic and cycloplegic SER by age, cycloplegic refractive error, axial length. The bias was greatest with younger age, hyperopic refractive error and shorter axial length. Differences approached zero when cycloplegic SER measures were myopic, and became increasingly negative with emmetropia and hyperopia (all *P* < 0.05, except for the high myopia subgroup, *P* = 0.491).

### 3.3 Machine learning model performance

With the clinical parameter values obtained from the development dataset, we developed and evaluated 6 ML models for predicting cycloplegic SER. In the development dataset, all 6 ML models predicted cycloplegic SER well with *R* ^*2*^ ranging from 0.935 (linear) to 0.966 (random forest), the correlation coefficient *r* of 0.952 (linear) to 0.973 (random forest), ME of −0.010 D (LightGBM) to −0.003 D (linear), and MAE of 0.292 D (random forest) to 0.405 D (linear) (Table 2). The random forest model performed best (*R* ^*2*^ = 0.966, MAE = 0.292 D), followed by LightGBM (*R* ^*2*^ = 0.956, MAE = 0.334 D). In the validation dataset, the MLP model outperformed others (*R* ^*2*^ = 0.934, *r* = 0.956, MAE = 0.385 D), with LightGBM model ranking second (*R* ^*2*^ = 0.931, *r* = 0.955, MAE = 0.387 D), and the random forest model performed worst (*R* ^*2*^ = 0.920, *r* = 0.951, MAE = 0.413 D) (Table 2). Paired *t*-tests confirmed no significant differences between predicted and measured SER across all models (*P* = 0.430 for linear, *P* = 0.927 for RF, *P* = 0.652 for XGBoost, *P* = 0.459 for MLP, *P* = 0.836 for SVM, *P* = 0.830 for LightGBM).

**TABLE 2:** Performance of Machine Learning models in the development and validation datasets.

Model	Development dataset (*n* = 1819 children, 3,638 eyes)	Validation dataset (*n* = 455 children, 910 eyes)
R^2^		
Linear	0.935	0.928
Random forest	0.966	0.920
XGBoost	0.954	0.929
MLP	0.948	0.934
SVM	0.945	0.930
LightGBM	0.956	0.931
ME, mean ± SD		
Linear	−0.003 ± 0.541	0.015 ± 0.557
Random forest	−0.005 ± 0.392	0.014 ± 0.584
XGBoost	−0.008 ± 0.456	−0.004 ± 0.551
MLP	−0.008 ± 0.484	−0.004 ± 0.533
SVM	−0.005 ± 0.495	0.002 ± 0.548
LightGBM	−0.010 ± 0.442	0.008 ± 0.544
MAE, mean ± SD		
Linear	0.405 ± 0.359	0.404 ± 0.383
Random forest	0.292 ± 0.262	0.413 ± 0.414
XGBoost	0.319 ± 0.326	0.391 ± 0.389
MLP	0.354 ± 0.330	0.385 ± 0.368
SVM	0.363 ± 0.338	0.389 ± 0.385
LightGBM	0.334 ± 0.290	0.387 ± 0.382

### 3.4 Subgroup analysis of the best-performing model

**FIGURE 2:**
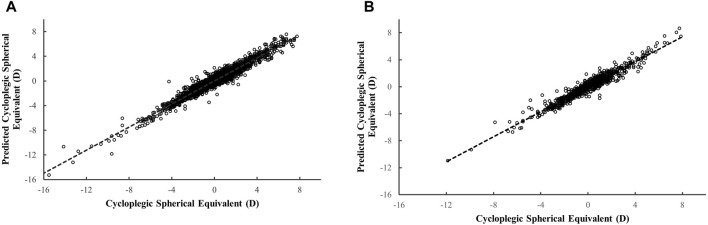
The scatter plot for the predicted versus measured cycloplegic spherical equivalent based on the MLP model. The scatter plot in the development dataset (**(A)** 3,638 eyes from 1819 children) and in the validation dataset (**(B)** 910 eyes from 455 children).

We further evaluated the performance of the best-performing ML model in the validation dataset (MLP) by assessing its performance across subsets stratified by age and cycloplegic SER magnitude. The importance of features in MLP model was shown in Supplementary Figure 1. Non-cycloplegic SER, AL/CR ratio, and AL were the top three predictors for predicting cycloplegic SER. The scatter plot (Figure 2) illustrated a highly positive correlation between predicted cycloplegic SER by MLP model and measured cycloplegic SER values, with *R* ^*2*^ of 0.948 in the development dataset and 0.934 in the validation dataset.

The overall ME between predicted and measured cycloplegic SER was −0.008 ± 0.484 D and −0.004 ± 0.533 D in two datasets (Table 3). In the development dataset, the ME was within 0.037 D and the MAE within 0.399 D across all age subgroups. When evaluating differences between predicted and measured based on magnitude of cycloplegic SER, the model exhibited the lowest MAE (≤0.328 D) for low myopia and emmetropia, and the highest MAE for high myopia and hyperopia > +2.00 D. Similar trends were observed in the validation dataset (Table 3).

**TABLE 3:** Subgroup analysis of the best-performing model MLP in the development dataset and validation dataset.

	Development dataset (n = 1819 Children, 3,638 eyes)					Validation dataset (n = 455 Children, 910 eyes)				
	Eyes	Mean error/D	Mean absolute error/D	Proportion of prediction errors≤±0.5 D/%	Proportion of prediction errors≤±1.0 D/%	Eyes	Mean error/D	Mean absolute error/D	Proportion of prediction errors≤±0.5 D/%	Proportion of prediction errors≤±1.0 D/%
Overall	3,638	−0.008 ± 0.484	0.354 ± 0.330	76.83	95.52	910	−0.004 ± 0.533	0.385 ± 0.368	73.08	94.29
By age/yrs										
3–6	1388	−0.020 ± 0.545	0.399 ± 0.371	72.41	93.59	348	0.010 ± 0.575	0.429 ± 0.382	67.24	91.95
7–10	2034	−0.004 ± 0.438	0.324 ± 0.294	79.70	96.76	508	−0.004 ± 0.499	0.353 ± 0.352	77.17	96.26
≥11	216	0.037 ± 0.490	0.345 ± 0.348	78.24	96.30	54	−0.095 ± 0.566	0.406 ± 0.402	72.22	90.74
By cycloplegic SER (D)										
≤-6.0	36	0.286 ± 0.931	0.606 ± 0.757	63.89	83.33	8	0.741 ± 0.913	0.831 ± 0.820	37.50	75.00
> −6.0 to ≤ −3.0	176	0.168 ± 0.540	0.390 ± 0.408	72.73	95.45	46	0.381 ± 0.745	0.529 ± 0.647	73.91	82.61
> −3.0 to ≤ −0.5	1369	0.093 ± 0.409	0.296 ± 0.296	83.56	97.22	343	0.060 ± 0.395	0.302 ± 0.261	83.09	98.25
> −0.5 to ≤+0.5	693	0.045 ± 0.436	0.328 ± 0.290	80.38	96.83	169	0.063 ± 0.489	0.371 ± 0.324	74.56	95.27
>+0.5 to ≤+2.0	956	−0.113 ± 0.460	0.370 ± 0.295	73.64	96.13	245	−0.117 ± 0.527	0.412 ± 0.348	64.9	96.33
>+2.0	408	−0.287 ± 0.593	0.517 ± 0.408	58.58	87.25	99	−0.297 ± 0.649	0.534 ± 0.472	58.59	80.81

To assess the clinical value of the ML model predictions of the refractive error, we evaluated the proportions of prediction errors within ±0.50 D and ±1.00 D compared with cycloplegic SER. The MLP model achieved clinical accuracy rates of 76.83% (±0.50 D) and 95.52% (±1.00 D) in the development dataset, and 73.08% (±0.50 D) and 94.29% (±1.00 D) in the validation dataset. Peak performance was observed in the subgroup of children aged 7–10 years (77.17∼79.70% within ±0.50 D) and for eyes with cycloplegic SER ranging from −3.00 to −0.50 D (83.09∼83.56% within ±0.50 D).

## 4 Discussion

This study developed and validated 6 ML models to predict cycloplegic refractive error using non-cycloplegic parameters in a large cohort of Chinese children. The results showed successful predictions of ML models in both the development and validation datasets, demonstrating the promising potential and practical value of ML-based tools to circumvent the challenges of cycloplegia in pediatric refractive assessments while maintaining diagnostic precision.

All the ML models performed well in predicting cycloplegic SER in the validation datasets (*R* ^*2*^ ranging from 0.920 to 0.934), with the MLP model performed best. Several studies have employed statistical prediction models to estimate cycloplegic refractive error in pediatric populations, utilizing diverse predictors such as demographics, ocular biometric parameters (Magome et al., 2021), non-cycloplegic refractive error (Sankaridurg et al., 2017; Wang et al., 2022), and uncorrected visual acuity [UCVA](Sankaridurg et al., 2017), alongside modeling methodologies ranging from traditional regression to advanced ML techniques. However, the heterogeneity in study designs and populations has resulted in widely variable predictive performance (*R* ^*2*^: 0.26∼0.935) (Ip et al., 2007; Kimura et al., 2007; He et al., 2015; Foo et al., 2016; Sankaridurg et al., 2017; Magome et al., 2021; Wang et al., 2022; Du et al., 2023; He et al., 2023; Ying et al., 2024). In particular, Du et al. (2023) developed 4 ML models (Support Vector Regression, Random Forest Regression, AdaBoost Regression, and Deep Neural Network) in 2,467 Chinese children aged 6–18 years, achieving *R* ^*2*^ of 0.899∼0.927 and MAE of 0.372∼0.436 D. Among them, AdaBoost regression model exhibited the best overall prediction performance, with *R* ^*2*^ = 0.927 and MAE = 0.372, slightly inferior to the optimal MLP-based model in our study. However, some predictors used in their models (e.g., the accommodation lag data) may not be easily available in clinical practice, which would limit the models’ applicability. Ying et al. (2024) also developed and validated 6 ML models based on 3,414 Chinese children ages 5–18 years by using age, gender, non-cycloplegic refractive error, biometric measures (AL, CR, AL/CR ratio, CCT, and anterior chamber depth), UCVA, intraocular pressure, and glasses-wearing status. The models predicted cycloplegic SER with *R* ^*2*^ of 0.913∼ 0.935, and MAE of 0.393∼0.480 D, with top performance comparable to ours. Notably, while the ML methods used in our study were largely similar to those employed by Ying et al., the optimal model diverged (MLP vs. XGBoost). This discrepancy may be attributed to cohort differences: their population comprised older children (5∼18 years), with nearly 50% was low hyperopia (+0.50∼+3.00 D); whereas ours encompassed younger ages (3∼16 years old), dominated by low myopia (−3.00∼-0.50 D) and low hyperopia (+0.50∼+2.00 D). In comparison to prior work, our study prioritizes clinical practicality by exclusively incorporating parameters easily obtainable without cycloplegia, while maintaining robust accuracy (MLP model: *R* ^*2*^ = 0.934). This balance between simplicity and precision positions our framework as a scalable solution for population-based studies, particularly in resource-limited settings where cycloplegic examinations are challenging. Furthermore, the included younger children (3–16 years old) with varied refractive status (from −16.13 to +7.88 D) enhances the model’s clinical relevance for early myopia risk stratification.

Substantial evidence demonstrates clinically significant discrepancies between cycloplegic and non-cycloplegic measurements, with non-cycloplegic SER systematically overestimated myopia (Zhao et al., 2004; Fotedar et al., 2007; Hu et al., 2015; Sankaridurg et al., 2017; Gu et al., 2022). Consistent with previous studies, such bias was also observed in our study: the mean SER changed from −0.04 ± 2.11 D to −0.43 ± 1.85 D under the non-cycloplegic condition with a mean difference of 0.39 ± 0.71 D toward myopia. Our systematic analysis of paired differences between cycloplegic and non-cycloplegic refraction across ages 3–16 years and all types of refractive error revealed that the overestimation of myopia or underestimated hyperopia with non-cycloplegia is greater in younger individuals, emmetropic or hyperopic eyes, and shorter AL. Accommodation significantly impacts the accurate measurement of refraction data in children. Age-related norms for accommodative responses in eyes without significant refractive error suggest that accommodative responses are quite variable under high accommodative demands, especially in younger individuals (McClelland and Saunders, 2004). Consequently, predicting refractive error without cycloplegia is challenging in young children. The inclusion of children under 5 years of age in this study provides a basis for addressing the difficulty of predicting cycloplegic refractive error in this age group.

The MLP model demonstrated robust clinical accuracy, with 73.08% and 94.29% of predictions falling within ±0.50 D and ±1.00 D of measured cycloplegic SER, respectively. This performance outperformed the traditional regression-based approach by He et al. (2023), which achieved an accuracy of 47% within ±0.50 D and 79% within ±1.00 D, using limited predictors (age, gender, AL and AL/CR ratio). While Du et al. (2023) reported marginally higher precision (75.2% within ±0.50 D) in their best-performing model, our framework maintains comparable efficacy while prioritizing clinically accessible parameters. Notably, the accuracy of MLP model peaked in children aged 7∼10 years (77.17∼79.70% within ±0.50 D) and those with low myopia (83.09∼83.56% within ±0.50 D). These subgroups represent pivotal stages for myopia surveillance and early intervention to mitigate progression to high myopia. These results position the ML model as a pragmatic tool for large-scale screenings in resource-limited settings.

There were several limitations in the present study. First, this study used the AB-800 optical biometer in a cohort of Chinese children, which may restrict the generalizability of the findings to other types of biometers or children of different races or ethnicities. External validation across diverse populations and devices is warranted. Second, the reduced accuracy in high myopia (≤−6.0 D) and moderate/high hyperopia (>+2.0 D) limits utility for these subgroups. Expanding data with extreme refractive errors and retraining models on enriched cohorts could improve performance and enhance the model’s applicability. Finally, the current ML models predict the cycloplegic refractive error at a single timepoint. Future longitudinal studies can evaluate the models’ capacity to track refractive progression over time.

## 5 Conclusion

This study developed and validated 6 ML models for predicting cycloplegic refractive error using readily available demographics and non-cycloplegic biometric parameters in a large cohort of Chinese children (3∼16 years). Among the evaluated algorithms, the MLP model demonstrated superior performance (*R* ^*2*^ = 0.934), and exhibited enhanced accuracy in children aged 7–10 years and those with low myopia—key populations for myopia surveillance and intervention. These ML models address practical challenges in pediatric refractive assessments, offering a scalable tool for clinical practice and large-scale epidemiological research where administering cycloplegia is not feasible.

## Data Availability

The raw data supporting the conclusions of this article will be made available by the authors, without undue reservation.
