# Supplementary figures and images for: Machine learning-driven prediction of cycloplegic refractive error in Chinese children
Source: Front Cell Dev Biol. 2025 May 22;13:1608494. doi: 10.3389/fcell.2025.1608494 (PMC12137252; doi:10.3389/fcell.2025.1608494)

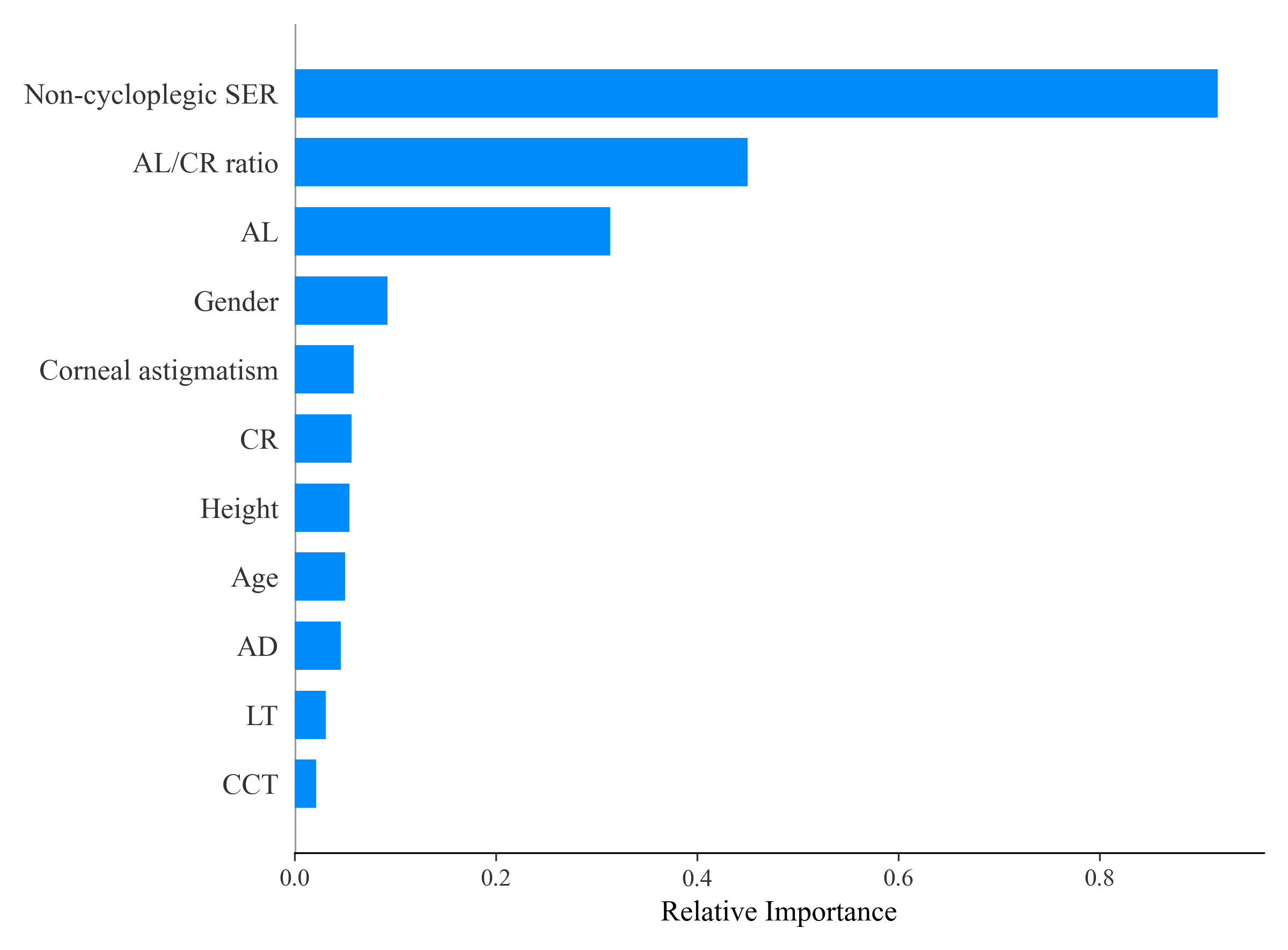

Supplement: Supplementary file 1 [file Image1.png]
